# A rare cause of acute compartment syndrome in the thigh: a case report

**DOI:** 10.1093/jscr/rjaa546

**Published:** 2020-12-28

**Authors:** Henry J T Slade, Koen De Ridder

**Affiliations:** Department of Orthopaedic Surgery, Wellington Regional Hospital, Wellington, New Zealand; Department of Orthopaedic Surgery, Wellington Regional Hospital, Wellington, New Zealand

## Abstract

Acute compartment syndrome (ACS) is an orthopaedic emergency that requires urgent fasciotomy and decompression to avoid significant morbidity. It is most commonly caused by a fracture or crush injury. We present a case of a patient who developed ACS of the posterior compartment of the thigh secondary to a low energy fall and avulsion of sclerotic arterioles. There was no fracture and the patient was not anti-coagulated. They had fasciotomy and embolization of responsible vessels. This case demonstrates the need for high clinical suspicion needed for ACS and the morbidity associated with a delayed fasciotomy. A literature research demonstrated no case reports of a patient developing ACS with no fracture, no crush injury and no history of anti-coagulation.

## INTRODUCTION

Acute Compartment Syndrome (ACS) is a complication of trauma, usually bleeding or oedema to a limb that compromises perfusion. The most common causes of ACS are a fracture to a limb or a crush injury [1]. The pathophysiology behind ACS is related to increasing pressure in a myofascial compartment. Due to the inelastic nature of the muscle and fascial surroundings, the increasing pressure causes venous hypertension in the compartment with associated tissue ischaemia [2]. As cell necrosis and lysis occurs osmotically, active cellular contents are released into the interstitial space, causing further fluid to gather and increasing pressure. The increased pressure causes arteriole blood supply to be cut off, leading to microvascular collapse [3]. Myonecrosis can occur in 2 hours in patients with ACS [4]. After 6 hours irreversible ischemia to muscle and neural tissues occurs within the affected compartment.

Diagnosis of this condition is difficult in the acute setting, as although ACS is well known amongst clinicians, no clear diagnostic criteria exist. The most common diagnostic symptoms and signs are pain out of proportion to the injury, pain on passive stretch of the muscle compartment and paraesthesia of sensory nerves within the compartment. Frustratingly for the clinician, these clinical signs in isolation have low sensitivity with ACS and also can occur in patients without ACS [5]. If you suspect ACS intramuscular compartment pressure (IMP), monitoring should be undertaken. If the IMP is within 30 mmHg of the patient’s diastolic blood pressure, this is the most sensitive test for ACS [6]. Unfortunately, there is often delay from assessment to diagnosis to surgical management [7]. Delays to treatment are associated with increasing morbidity for the patient [8].

Treatment for ACS remains surgical fasciotomy release. This allows the muscle compartment to expand and the pressure to be released. The general consensus is that performing a fasciotomy on a suspected ACS patient is better than a late fasciotomy or missed ACS. This is due to the risks associated with missed or late ACS such as rhabdomyolysis and muscle necrosis as well as significant functional loss.

We present a patient who was admitted to our hospital after a fall from standing height who developed ACS in their posterior compartment of thigh. This was an unusual presentation, as there was no fracture, no crush injury and the patient was not on anti-coagulative medication. There are no case reports in the literature of patients who have developed ACS without any of the three aforementioned risk factors.

## CASE REPORT

This female patient in her 50s presented to the emergency department (ED) via ambulance at 03:30 am. She has a past medical history of obesity and gastrooesophogral reflux disease and is a non-smoker. She had slipped on the floor and landed onto her left side at 11:30 pm. She was unable to get up from the floor due to significant pain in her left leg. Her husband found her 2 hours later and rang an ambulance. The paramedics administered significant amounts of analgesia during transit including intra-muscular Ketamine, Morphine and inhaled Methoxyflurane.

Initial assessment by an ED doctor noted the patient keeping their left hip in flexed position with tense palpable haematoma in the posterior-medial aspect of the left thigh. Noted that the patient was confused due to ketamine use and so could not ascertain if could actively move their toes or test for sensation in the foot. Capillary refill was <2 seconds distally with palpable pulses.

Provisional diagnosis was a femoral shaft fracture. X-ray was taken and demonstrated no evidence of femoral fracture. The patient was re-reviewed at 07:00 am with ongoing severe pain, paraesthesia in L5/S1 dermatomes and myotomes of the foot and referred to orthopaedics for urgent review.

**Figure 1:**
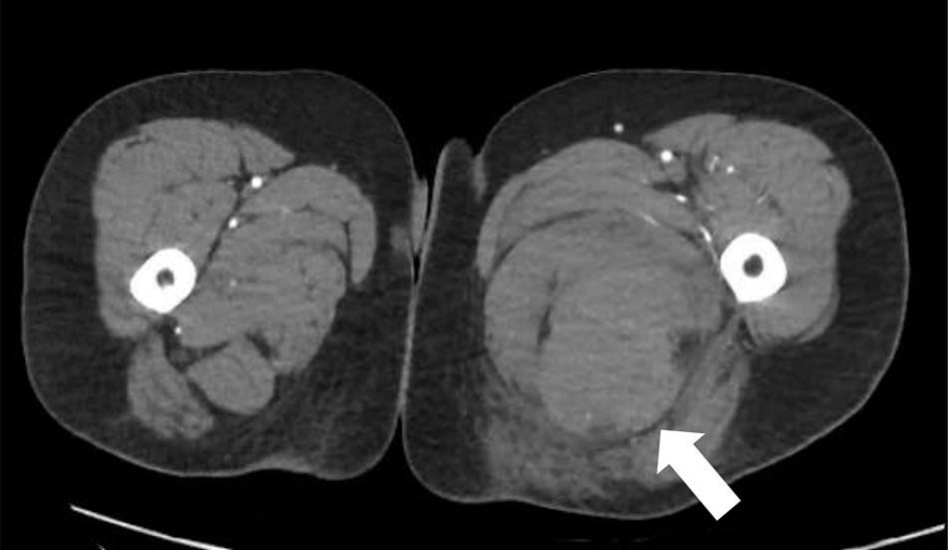
CT angiography axial slice demonstrating haematoma in posterior compartment of left thigh (white arrow).

The patient was reviewed on ward around at 7:30 am by consultant orthopaedic surgeon. An urgent computed tomography (CT) angiography of the affected side was performed, as it was unclear if the haematoma was in the posterior or medial compartment; this demonstrated a large haematoma in the posterior compartment, with no cause identified (Fig. 1).

The patient was taken to theatre for fasciotomy at 11:00 am. Medial incision over the thigh once incised through the posterior compartment fascia a large haematoma was evacuated. Semi-membranous was 100% ruptured and the subsequent cavity extended proximally to the posterior proximal thigh.

No clear bleeding point could be identified, so decision was made to close the skin with a corrugated drain left *in situ*. Following closure, there was significant bleeding through the dressing and so the wound was re-explored with vascular surgical input. Bleeding was identified from the proximal extent of this cavity, which could not be accessed surgically. The patient was subsequently taken to angiography for identification of bleeding point and embolisation.

**Figure 2:**
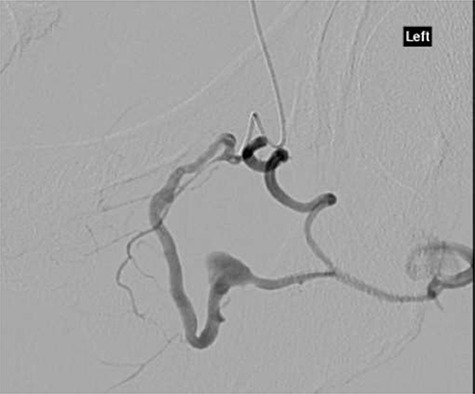
CT angiography with dye showing dilatation of collateral branch of left obturator artery.

**Figure 3:**
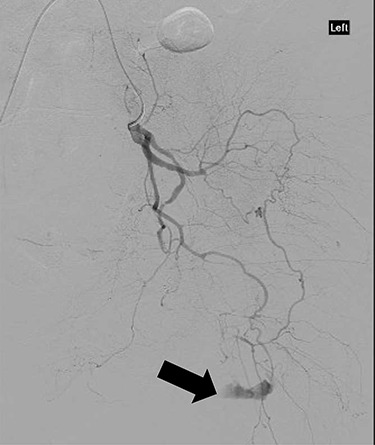
CT angiography showing extravasation of dye from muscular branch of the internal iliac artery (black arrow).

CT angiography showed multiple hypertrophied branches of the left internal iliac artery with heavy collateralisation to the territory of occluded left common femoral artery. Pathological dilatation of the collateral branch of the left obturator artery was identified as source of bleeding and embolized (Fig. 2). A further source of bleeding from a collateral muscular branch of the internal iliac artery was also embolized (Fig. 3).

During this time the patient lost 4 litres of blood and so had multiple transfusions, she developed an acute kidney injury and was managed on the intensive care unit for 2 days.

A total of 3 days later, she had her thigh wound closed. She developed a postoperative non-infected seroma, which was drained. At the time of writing, she is still left with a sciatic nerve injury related to compression. She has no sensation in L5/S1 dermatomes. Medical Research Council power scale 0/5 in L5 and S1 myotomes. She is able to mobilise with an ankle and foot orthosis to prevent her foot drop and will be followed up in the hope that function returns.

## DISCUSSION

This case demonstrates the difficult clinical diagnosis of ACS and the importance of having high clinical suspicion especially in the context of severe pain out of proportion. It also demonstrates the importance of a timely diagnosis and progression to surgical fasciotomy with decompression. The surgery was nearly 12 hours postinjury. The patient has been left with morbidity such as neurological pain and a foot drop.
